# Current Role of Intra-Articular Injections of Platelet-Rich Plasma in Adhesive Capsulitis of Shoulder: A Systematic Review

**DOI:** 10.3390/bioengineering10010021

**Published:** 2022-12-22

**Authors:** Bushu Harna, Vijay Gupta, Shivali Arya, Naveen Jeyaraman, Ramya Lakshmi Rajendran, Madhan Jeyaraman, Prakash Gangadaran, Manish Khanna, Chae Moon Hong, Byeong-Cheol Ahn

**Affiliations:** 1 Indian Orthoapedic Rheumatology Association (IORA), Lucknow 226010, Uttar Pradesh, India; 2 Indian Stem Cell Study Group (ISCSG) Association, Lucknow 226010, Uttar Pradesh, India; 3 Department of Orthopaedics, Max Super Speciality Hospital, Patparganj, New Delhi 110017, India; 4 Elite Clinic for Orthopaedics and Critical Care Centre, Delhi Gate Agra 282002, Uttar Pradesh, India; 5 Department of Radiodiagnosis, Post Graduate Institute of Medical Education and Research, Chandigarh 160012, India; 6 Department of Orthopaedics, Rathimed Speciality Hospital, Chennai 600040, Tamil Nadu, India; 7 Department of Nuclear Medicine, School of Medicine, Kyungpook National University, Kyungpook National University Hospital, Daegu 41944, Republic of Korea; 8 Department of Orthopaedics, ACS Medical College and Hospital, Dr. MGR Educational and Research Institute, Chennai 600056, Tamil Nadu, India; 9 BK21 FOUR KNU Convergence Educational Program of Biomedical Sciences for Creative Future Talents, Department of Biomedical Sciences, School of Medicine, Kyungpook National University, Daegu 41944, Republic of Korea

**Keywords:** periarthritis shoulder, platelet-rich plasma, adhesive capsulitis, frozen shoulder, steroid

## Abstract

Adhesive capsulitis shoulder is a common problem of patients presenting with shoulder pain and disability. The approach to such patients includes a variety of modalities. This systematic review evaluates the efficacy of intra-articular injections of platelet-rich plasma (PRP) in the treatment. A literature search was performed between January 2010 and 30 May 2022. MeSH terms used were ‘Platelet-rich plasma’ OR ‘PRP’ AND ‘Frozen shoulder’ OR ‘Adhesive capsulitis shoulder’ OR ‘Periarthritis shoulder’. The search included published articles in the English language involving human subjects. Studies evaluating other types of shoulder disorders, in vitro studies, review articles, animal-model studies, and pre-clinical trials were excluded. The data regarding study characteristics, efficacy, and safety outcomes were analyzed. A total of 11 studies with 347 patients over 10 years were finally included in this review. Most publications were in 2019 and 2020, mostly from India. This review included seven comparative studies, three case series, and one case report. In seven studies, a single intra-articular PRP injection was administered, whereas in the rest of the studies two or multiple injections were given. Only one study demonstrated an equivocal efficacy of PRP and steroid intra-articular injection. The rest all depicted better clinical and functional outcomes with the PRP injection. Only one study compared the outcomes of hydro-dissection treatment in adhesive capsulitis with the intra-articular PRP injection. The rest all either examined PRP alone or compared it with the steroid intra-articular injection. None of the studies showed any major side effects. The intra-articular injections of PRP in the management of adhesive capsulitis of the shoulder provide a new treatment approach. Further studies are required to ascertain the efficacy and safety of the PRP intraarticular injection as a management alternative in adhesive capsulitis.

## 1. Introduction

Adhesive capsulitis, more commonly known as frozen shoulder is one of the most common clinical shoulder syndromes. It usually affects the middle-aged and geriatric population [1]. There are a few predisposing factors such as diabetes mellitus, thyroid disorder, rotator cuff injury, cardiovascular diseases, etc., and it is more frequent in some populations and geographical areas [2,3]. The pathophysiology of frozen shoulder is not fully understood, but various mechanisms have been proposed. The inflammatory changes, fibrosis, and capsular contracture are part of the pathological changes [4]. The disease is self-limiting but very disabling. The pain, especially night pain and shoulder stiffness, are worrisome symptoms. The treatment of adhesive capsulitis ranges from physical and rehabilitative medicine and medications to intra-articular injections and, rarely, surgery [5]. A multimodal approach is generally required to relieve the symptoms. Most of the patients can be managed with conservative management, including analgesic drugs and physical therapy. However, there are a few groups of patients unresponsive to conservative treatment or who have a recurrence of the disease, which leads to the implementation of invasive therapies such as intraarticular steroid injections, hydro-dissection, and, rarely, arthroscopic surgical release. All these modalities, however, do not address the pathophysiology but give symptomatic relief. The injection of platelet-rich plasma (PRP) intra-articularly gives long-term resolution and prolonged efficacy, as it resolves the etiopathological changes [6]. This review evaluates the role of PRP, assessing the safety and efficacy in the treatment of adhesive capsulitis shoulder.

## 2. Materials and Methods Search Strategy

**Figure 1:**
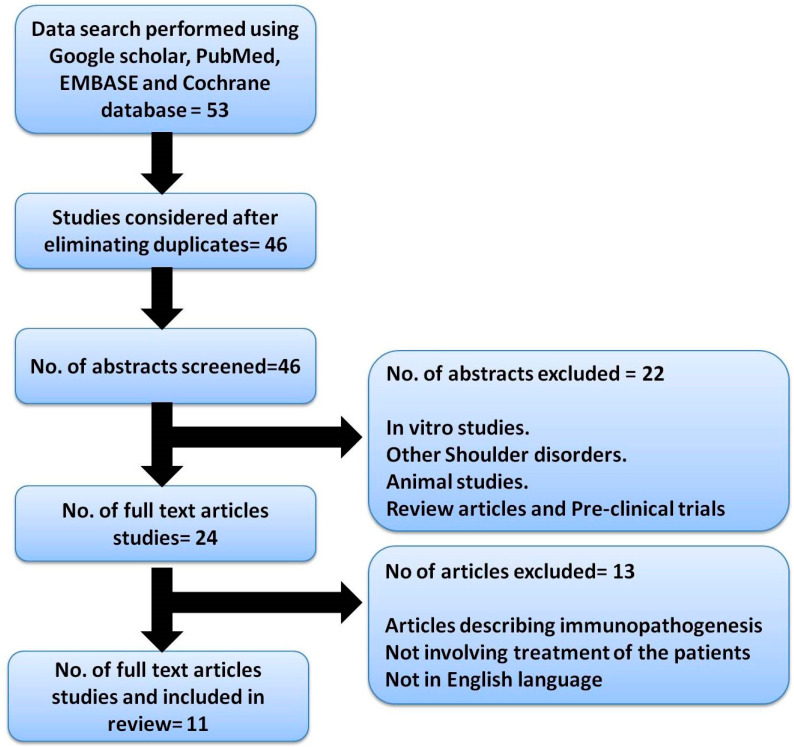
Describing the PRISMA chart.

A data search was performed by using online databases for all the studies published on human subjects in the English language between January 2010 and May 2022. The databases included in the search were Google Scholar, PubMed, Cochrane database, and EMBASE, and the review report was based on PRISMA guidelines with registered no INPLASY2022110127 as depicted in Figure 1. 

The terms used for an independent electronic search by the two researchers were ‘Platelet-rich plasma’ OR ‘PRP’ AND ‘Frozen shoulder’ OR ‘Adhesive capsulitis shoulder’ OR ‘Periarthritis shoulder’. Any discrepancy between the authors was resolved with the help of the senior author.

*Inclusion criteria*: Human adult patients with diagnosed adhesive capsulitis/frozen shoulder undergoing treatment with the help of PRP injections, in vivo studies, and articles published after the year 2010 (the last 12 years).

*Exclusion criteria*: Studies including patients suffering from other shoulder disorders (including trauma, rotator cuff injury, etc.), in vitro studies, review articles, animal model studies, and pre-clinical trials were excluded. Articles primarily evaluating only immunopathogenesis or the process of the action of platelet-rich plasma/PRP were also excluded.

### Data Extraction

The search showed literature published in various forms, including clinical trials, original studies, case series, and case reports, all of which were considered. The review was performed by the two authors in consultation with the senior author, independently. The independent decision of the senior author was considered final. The data from the literature search was extracted from Microsoft Excel. Various characteristics of the data extracted were analyzed, including (i) author, type of study, number of patients enrolled in the study, place and year of publication, (ii) efficacy outcomes, and (iii) safety outcomes: adverse events, as mentioned in Table 1. The details of the isolation protocols of PRP are mentioned in Table 2.

Statistical analysis could not be carried out because of the heterogeneity of the studies included in the review.

## 3. Results

The electronic database search resulted in 46 studies. The title and abstract of all these articles were analyzed for inclusion in this review. The data not available in the abstract was searched for in the full paper, and if not available the study was excluded. After considering the inclusion criteria and removing the duplicates, 11 studies were finally included in this review. A PRISMA (Preferred Reporting Items for Systematic Reviews and Meta-Analyses) flowchart depicting the literature search is presented (Figure 1).

A total of 347 patients were administered the PRP injection. Over 10 years, one study was published in 2015 and one in 2017, two studies in 2018, four studies in 2019, and three in 2020. Most publications were from India, followed by Turkey, Iran, and China. This review included seven comparative studies, three case series, and one case report.

The case report involves two PRP injections, 1 month apart. In one case series, the PRP injection was given in two sessions, whereas in another a single PRP injection only was administered. One case series administered two PRP injections 2 weeks apart, whereas the other case series administered a single dose of the intra-articular PRP injection only.

Only one comparative study evaluated the procaine injection and the injection of PRP in the frozen shoulder. One randomized study compared the efficacy of the PRP injection given 3 times every 2 weeks with the saline injections given intra-articularly at the same frequency and volume.

The three randomized clinical studies compared the intra-articular corticosteroid injection with the PRP injection. Methylprednisolone was the corticosteroid injection used. One study used an injection of lignocaine with both corticosteroid and PRP injections intra-articularly. One study evaluated the effectiveness of the PRP injection on the control study population who were given normal saline intra-articularly.

One study subdivided the study population into three groups, comparing the effectiveness of the intra-articular PRP injection, intra-articular corticosteroid injection, and ultrasonic therapy.

Only one study compared the outcomes of hydro-dissection treatment in adhesive capsulitis with the intra-articular PRP injection.

Nevertheless, all studies but one depicted better clinical and functional outcomes with the PRP injection. In seven studies, only one intra-articular PRP injection was administered, and in the rest of the studies, two or multiple injections were given. None of the studies showed any major side effects.

## 4. Discussion

The present study reviewed the literature on the management of adhesive capsulitis shoulder, assessing the efficacy and safety of PRP. Adhesive capsulitis is a complex shoulder disease with varied etiopathogenesis. The pathophysiological changes include inflammation, reactive fibrosis, and adhesions of the synovial membrane in the shoulder joint [18]. This may be depicted by subacromial fibrosis, proliferative synovitis, and capsular thickening. The disease is self-limiting but very disabling and has varied and long resolving times. The disease typically occurs in three phases: phase I—the ‘freezing’ painful phase; phase II—the ‘frozen’ or stiff phase; phase III—the ‘thawing’ or recovery phase. The duration of phase I varies, from 2 to 9 months, phase II can last for 4 to 12 months, and lastly, phase III can last for 1 to 3 years. Even after the recovery phase, there can be permanent symptoms in up to 40% of the patients [2,19]. There are various risk factors such as thyroid disorder, diabetes mellitus, other endocrinopathies, and trauma, which can lead to the occurrence of adhesive capsulitis at a young age, and a bilateral adhesive capsulitis with a longer recovery time, and sometimes a reoccurrence of the disease.

There are various treatment modalities described to manage this condition. The treatment is aimed at alleviating the pain and restricted movement of the shoulder joint. A multimodal approach is required in the management. These treatments include analgesics, physical and rehabilitative medicine consisting of stretching exercises, minimally invasive intra-articular injections, hydro-dissection, manipulation under anesthesia, invasive procedures such as arthroscopic or open synovectomy, and capsular releases. These can be utilized alone or in combination for better management of adhesive capsulitis shoulder. All these treatments provide symptomatic relief in the patients and do not affect the pathoanatomy of the disease much.

Recently, PRP has been investigated in the treatment of adhesive capsulitis. PRP, as the name suggests, is a concentrate of platelets in plasma, which degranulates the alpha granules and which further contains various growth factors such as platelet-derived growth factor (PDGF), vascular endothelial growth factor (VEGF), transforming growth factor-beta (TGF-β), fibroblast growth factor (FGF), hepatocyte growth factor (HGF), and epithelial growth factor (EGF), and the cytokines that help in the healing of soft tissue [20]. In the literature, the isolation of PRP by various methods remains the subject of debate and a mystery, as it is a personalized product when used with autologous PRP. Hence, optimal standardization is required to maintain the quality of PRP to be obtained and injected for various diseases. PRP is obtained using the differential centrifugation method, by obtaining a patient’s whole blood. Approximately 10 mL of venous blood is drawn and subjected to the first centrifugation [3000 rpm (approx. 302× *g*) for 10 min] and the resultant plasma is subjected to the second centrifugation [5000 rpm (approx. 839× *g*) for 10 min]. PRP is injected into the shoulder joint, along with the activator such as 10% calcium gluconate in the ratio of 10:1. The supraphysiological release of growth factors from the PRP helps in neovascularization, the synthesis of collagen, and the activation of the resident stem-cells [21]. These growth factors heal the injured tissue by reducing inflammation, followed by the proliferation and remodeling of the reparative tissue [22]. The molecular basis of platelet-rich plasma is due to increased HGF and TNF-α activity caused by disrupting the NF-κB transactivating activity. PRP therapy has depicted tissue healing in various tissue lesions without major complications such as tendon lesions, tendinopathy, muscle tears, ligament tears, or even fractures. The role of PRP has already been investigated in the management of rotator cuff tears, with promising results [23].

PRP was compared to procaine in the treatment of adhesive capsulitis shoulder in a randomized study by Lin et al. [16] The study included 60 patients, divided into two groups. One group was given 2 mL PRP and another group was given procaine, while the rest of the physical and rehabilitative medicine and assessment were the same for both groups. There was a reduction in the visual analog score (VAS) and linear improvement in the University of California Los Angeles (UCLA) scores for the PRP group. The study depicted better and longer efficacy than for the procaine group. In a study involving 44 frozen-shoulder patients by Aslani MA et al. [7], PRP was given in two stages. In stage one, one PRP injection was injected into the subacromial bursa and the intra-articular space. The PRP injections were repeated after 4 weeks, at the same site, whereas in stage two only the glenohumeral joint was injected with the PRP injection. The results showed a significant reduction in pain (*p* ˂ 0.001), and improvement in the range of motion of the shoulder and function scores, at the follow-up of 25 weeks. In the study, 66.7% of the patients reported an improvement in pain, 51.6% of patients depicted improvement in disabilities of the arm, shoulder, and hand (DASH) score, and 100% in the short-form-survey (SF-12) health questionnaire, with 65% of the patients satisfied with the treatment protocol. The results were encouraging for the use of PRP injections as a safe and non-surgical intervention for decreasing pain and improving shoulder mobility in adhesive capsulitis shoulder. The case report by Aslani H et al. [17] reported a 45-year-old gentleman with a frozen shoulder managed with two consecutive intra-articular PRP injections given in the seventh and eighth month after the initiation of symptoms. There was improvement regarding diurnal shoulder pain, DASH score, shoulder movement, no night pain, and a reduction in VAS scores. Feusi et al. [24] demonstrated the histological improvement in the in vivo shoulder-contracture model in rats. The PRP showed a reduction in severity grade in the tissue histology of some parts of the frozen shoulder.

A study by Unlu et al. [9] included 32 patients with adhesive capsulitis, randomized into two groups. One group was given PRP injections 3 times every 2 weeks, while in the control group saline injections with the same volume and at the same frequency were injected into the shoulder joint. The PRP group depicted improvement in the shoulder-pain-and-disability-index (SPADI) score and range of motion, along with a decrease in the VAS score. The study concluded that the PRP injection was efficacious in alleviating pain and improving shoulder joint motion in adhesive capsulitis patients.

In the study by Agrawal et al. [11], 20 adhesive-capsulitis-shoulder patients treated with a single injection of 4 mL PRP were evaluated. The patients were followed up for a month and assessed using the Constant–Murley shoulder score. There was a statistically significant improvement in Constant–Murley shoulder scores at the end of 1 month, though there was an increase in pain and decreased shoulder movement in a few patients on the third day. No complications warranting treatment were seen in any of the patients. The results demonstrated that even a single PRP injection in adhesive capsulitis improved shoulder motion and decreased pain.

In a study [12] including nine adhesive-capsulitis patients managed with two ultrasound-guided glenohumeral-joint PRP injections given two weeks apart, there was significant improvement depicted in VAS scores and SPADI scores at 2, 6, and 12 weeks (*p* < 0.05). A similar improvement was seen in the active and passive range of motion at weeks 2, 6, and 12 (*p* < 0.05). The case series reported PRP as an alternative treatment for adhesive-capsulitis-shoulder patients.

A few comparative studies assessing the efficacy of the intra-articular injection of PRP and corticosteroid injection have been conducted. In an interesting study by Kumar et al. [13], the efficacy of intra-articular corticosteroid injections with PRP in the treatment of adhesive capsulitis was compared. Sixty patients included in the study were divided into two groups. A single injection of PRP and 2 mL (80 mg) methylprednisolone with 6 mL of 2% lignocaine hydrochloride was injected into the shoulder joint via the posterior approach, respectively. The results depicted no difference in the efficacy of PRP and steroids in the management of adhesive-capsulitis-shoulder patients during the follow-up at 6 months. In the study by Upadhyay et al. [10], 120 adhesive-capsulitis-shoulder patients were studied in a prospective randomized assessor-blind comparative analysis. All the patients were given an intra-articular injection of either methylprednisolone (steroid) or PRP with 1% lidocaine. The study showed the PRP injections to be more efficacious and with more sustained effects than the corticosteroid injection, at the follow-up at 6 months. The PRP group showed linear improvement in the mean pain scale, mean disability, and total SPADI score. The scores for PRP were higher at the follow-up at 1 and 3 months, compared with the steroid groups (*p* < 0.05). The study concluded the potential and longer sustained efficiency of the PRP injection in improving functional outcomes and helping to decrease pain.

In a cohort study by Barman et al. [8], single intra-articular PRP and corticosteroid injections were comparatively evaluated in the management of adhesive capsulitis shoulder. In the group of 30 patients, an intra-articular single injection of 2 mL (40 mg) of methylprednisolone acetate mixed with 2 mL 2% lignocaine was given via the posterior approach, under the ultrasound guidance, whereas in the other group, 4 mL of single-centrifugation PRP was injected. The PRP group consisted of 28 patients, whereas the steroid group had 27 patients who were followed for the next 12 weeks. There was a significant decrease in the VAS score and an increment in the SPADI score among the patients in the PRP group. There was a statistical improvement in passive abduction and external rotation in the PRP group, as compared to the steroid-group patients. No major adverse effects were reported by any patient. The study concluded that a single dose of intra-articular PRP injection was more efficacious than corticosteroid at the follow-up at the end of 12 weeks. There was an improvement in the range of movement of the shoulder and a reduction in pain and disability in adhesive-capsulitis-shoulder patients.

In the study by Kothari et al. [14], the efficacy of PRP, corticosteroid injection, and ultrasonic therapy in the adhesive capsulitis shoulder were comparatively evaluated. The study population of 195 adhesive-capsulitis-shoulder patients was randomly divided into three groups. One group was managed with a single intraarticular injection of 2 mL PRP, the other group was treated with 80 mg of intra-articular methylprednisolone injection and the third group was managed with ultrasonic therapy only (seven times in two weeks; 1.5 W/cm^2^, 1 MHz, continuous mode). At the 12-week follow-up, a statistically significant improvement was seen in the PRP group with active as well as the passive range of shoulder motion, and the VAS and QuickDASH scores when compared with the corticosteroid or ultrasonic-therapy group. Even at 6 weeks, the PRP group showed statistically significant improvements in VAS and QuickDASH ultrasonic therapy. No complication was observed in the study. This study reported the efficacy of a single PRP injection over the corticosteroid or ultrasonic-therapy group in the management of adhesive capsulitis shoulder.

The study by Jeyaraman et al. [15] compared PRP and hydro-dissection in the treatment of adhesive capsulitis shoulder. The study population included 100 patients, who were divided equally into two groups. Group A patients received an autologous PRP injection and group B patients received a hydro-dissection of the shoulder. All the patients were evaluated before and after the procedure for improvement in the motion of the shoulder and pain relief. The statistical analysis for 46 patients and 45 patients in groups A and B, respectively, showed a statistical improvement in the range of motion of the shoulder and better pain relief in group A patients (*p* < 0.001 for VAS score and *p* < 0.01 for DASH score). Autologous PRP therapy improves the function of the shoulder, along with the movement, thus affecting the functional quality of life with long-term outcomes. The autologous PRP therapy remains functionally superior to hydro-dissection, by rejuvenating the degenerative tissues.

Intra-articular PRP administration offers a promising new technique in the management of adhesive capsulitis, although further randomized-control trials and meta-analyses are required to ascertain the safety and efficacy of PRP. The technique of preparing PRP varies among the authors and requires a standardization protocol to maintain the quality of PRP.

Limitations of the study: This systematic review does not include review articles and in vitro studies. Only human studies are included. The statistical analysis of the studies could not be performed, due to the heterogeneity of the studies. Case reports, comparative analyses, and randomized-control studies are part of this review. The preparation and quality of the PRP were not described in all the articles.

Further larger multicentric studies are required to assess the efficacy of PRP in the treatment of adhesive capsulitis shoulder.

## 5. Conclusions

PRP in the management of adhesive capsulitis of the shoulder provides a new avenue of treatment, tackling the disease at the pathophysiological level, although efficacy and safety are yet to be established. PRP shows better results than other modalities of conservative management in reducing pain and improving movement in adhesive-capsulitis-shoulder patients. Further research is required to ascertain and establish PRP intraarticular injection as a management alternative in adhesive capsulitis.

## Figures and Tables

**Table 1:** Describing the details of various studies.

S.No	Author	Year	Type of Study	Country	No of Patients	Study Details	Results	Complications
1.	Aslani MA et al. [7]	2020	Case series	Iran	44	First stage: one PRP injection in each subacromial bursa and intra-articular space, repeated after 4 weeks. Second stage: one PRP injection only, in the glenohumeral joint.	Significant improvement in function: 51.6% in DASH and 100% in SF-12 health-survey questionnaire. Significant reduction in pain function (*p* < 0.001), 67% improvement in pain. Follow-up: 25 weeks.	None
2.	Barman et al. [8]	2019	Comparative study	India	28	A total of 28 patients were given single intra-articular PRP, and 27 patients given single intra-articular corticosteroid.	PRP group: reduction in VAS score and total SPADI score, compared with corticosteroid group. Significant improvement in ROM in the PRP group. Follow-up: 12 weeks.	None
3.	Unlu et al. [9]	2020	Randomized controlled trial	Turkey	17	Randomized into two groups: one group was given a 2 mL PRP injection three times every two weeks, while the control group was given a saline injection Intra-articularly. The same exercises were given to both groups.	Improvement in SPADI score and ROM in the PRP group, as compared to the control group. Significant decrease in VAS score in PRP group. There is no significant difference in the use of analgesics in both groups.	None
4.	Upadhyaya et al. [10]	2020	Randomized comparative analysis	India	60	Patients received a single injection, either methylprednisolone (Group II) or PRP (Group I) with 1% lidocaine. The patients were randomized into these groups.	The mean pain scale, mean disability, and total SPADI score linearly improved in the PRP group, and final scores at the final follow-up were higher, compared with the steroid group (*p* < 0.05).	None
5.	Agarwal et al. [11]	2019	Case series	India	20	Patients were given a single 4 mL PRP injection.	At 1 month, statistically significant improvements in the constant score. A few patients had increased pain on the third day of injection, which improved with time.	None
6.	Kumar et al. [12]	2019	Comparative study	India	30	Patients were divided into two groups: one received the PRP injection, whereas the other was given methylprednisolone.	A significant difference in VAS between 1 month, 2 months, and 6 months. A significant difference in VAS between 2 months and 6 months. The outcome was excellent amongst 10% of subjects, good amongst 66.67% of subjects, fair amongst 13.33% of subjects, and poor amongst 10% of patients. Both PRP and steroids showed equal effectiveness in treating frozen shoulders.	None
7.	Calis et al. [13]	2019	Case series	Turkey	9	One intra-articular PRP injection was given at presentation and 2 weeks. PRP injection was given under sonography guidance.	Significant improvements in ROM (*p* < 0.05), VAS scores (*p* < 0.05) and SPADI (*p* < 0.05) at the follow-up of 12 weeks.	None
8.	Kothari et al. [14]	2017	Randomized comparative study	India	62	Patients were divided into the A, B, and C groups, and were given corticosteroid 80 mg, PRP(2 mL), and ultrasonic therapy (seven times in 2 weeks).	PRP injection depicted better results in terms of ROM, VAS score and QuickDASH score during the corticosteroid and ultrasonic therapy. Follow-up: 12 weeks.	None
9.	Jeyaraman et al. [15]	2018	Comparative study	India	46	Group A: given 3 mL PRP intra-articularly. Group B: hydro-dissection (20 mL normal saline + 5 ml lignocaine) intra-articularly.	PRP group showed improved functional quality of life. Significant improvement in ROM (DASH score *p* < 0.01) and decrease in VAS score (*p* < 0.01).	None
10.	Lin et al. [16]	2018	Randomized control trial	China	30	Group 1: 2 mL PRP intra-articularly. Group 2: Procaine injection intra-articularly.	VAS score decreased in both groups till 3 months but continued to decline in the PRP group whereas increased in the procaine group. The functional improvement (UCLA score) improved in the PRP group.	None
11.	Aslani H et al. [17]	2016	Case report	Iran	1	PRP injections are given in the seventh and eighth month after the start of symptoms.	After the first injection: improvement was seen regarding diurnal shoulder pain and night pain. Improvement in ROM and function.	None

**Table 2:** Details of isolation protocols of PRP in the included studies.

S.No	Author	Year	PRP System Used	Type of PRP Used	Spin Technique
1.	Aslani M A et al. [7]	2020	Arthrex-ACP system	Not specified	1500 rpm for 5 min
2.	Barman et al. [8]	2019	Plasmamed PRP Kit	Not specified	1800 rpm for 14 min
3.	Unlu et al. [9]	2020	Manual method	Not specified	1200 g for 5 min
4.	Upadhyaya et al. [10]	2020	Manual method	Not specified	1500 rpm for 15 min
5.	Agarwal et al. [11]	2019	Manual method	LR-PRP	Not specified
6.	Kumar et al. [12]	2019	Not specified	Not specified	Not specified
7.	Calis et al. [13]	2019	Manual method	LP-PRP	1195 rpm for 20 min followed by 1890 rpm for 15 min
8.	Kothari et al. [14]	2017	Manual method	Not specified	Not specified
9.	Jeyaraman et al. [15]	2018	Manual method	Not specified	3000 rpm for 10 min followed by 5000 rpm for 10 min
10.	Lin et al. [16]	2018	Manual method	Not specified	Not specified
11.	Aslani H et al. [17]	2016	Arthrex-ACP system	Not specified	5000 rpm for 5 min

## Data Availability

The data presented in this study are available on request from the corresponding author.
